# Evolution of the Translocation and Assembly Module (TAM)

**DOI:** 10.1093/gbe/evv097

**Published:** 2015-05-20

**Authors:** Eva Heinz, Joel Selkrig, Matthew J. Belousoff, Trevor Lithgow

**Affiliations:** ^1^Department of Microbiology, Monash University, Melbourne, Victoria, Australia; ^2^Department of Biochemistry & Molecular Biology, Monash University, Melbourne, Victoria, Australia; ^3^Present address: European Molecular Biology Laboratory, Genome Biology Unit, Heidelberg, Germany

**Keywords:** TamA, TamB, beta-barrel assembly, translocation, outer membrane, membrane biogenesis

## Abstract

Bacterial outer membrane proteins require the beta-barrel assembly machinery (BAM) for their correct folding and function. The central component of this machinery is BamA, an Omp85 protein that is essential and found in all Gram-negative bacteria. An additional feature of the BAM is the translocation and assembly module (TAM), comprised TamA (an Omp85 family protein) and TamB. We report that TamA and a closely related protein TamL are confined almost exclusively to *Proteobacteria* and *Bacteroidetes*/*Chlorobi* respectively, whereas TamB is widely distributed across the majority of Gram-negative bacterial lineages. A comprehensive phylogenetic and secondary structure analysis of the TamB protein family revealed that TamB was present very early in the evolution of bacteria. Several sequence characteristics were discovered to define the TamB protein family: A signal-anchor linkage to the inner membrane, beta-helical structure, conserved domain architecture and a C-terminal region that mimics outer membrane protein beta-strands. Taken together, the structural and phylogenetic analyses suggest that the TAM likely evolved from an original combination of BamA and TamB, with a later gene duplication event of BamA, giving rise to an additional Omp85 sequence that evolved to be TamA in *Proteobacteria* and TamL in *Bacteroidetes*/*Chlorobi*.

## Introduction

**Fig. 1.—:**
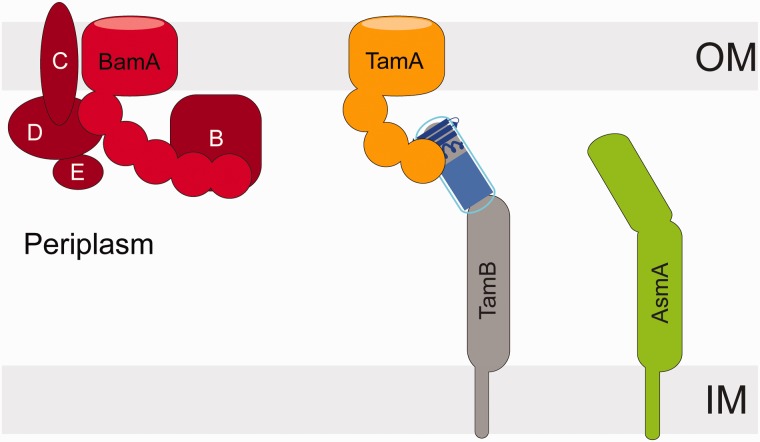
The BAM and the TAM in *E. coli*. The two Omp85 proteins BamA and TamA are membrane-embedded beta-barrels, with their POTRA domains extending into the periplasm. In *E. coli*, BamA interacts with four lipoprotein partners: BamB, BamC, BamD, and BamE to form the BAM complex. The TAM is formed from TamA in the outer membrane and the innermembrane anchored protein TamB. Sequence analysis suggests a relationship between the TamB protein family and the AsmA protein family. In *E. coli*, the inner membrane protein AsmA is not known to interact with any outer membrane protein partner.

Bacterial outer membrane proteins show a range of complexity in their domain arrangements, ranging from comparatively simple porins to large and complex molecules such as autotransporters, intimins, and invasins. The canonical structure for the membrane-embedded domain of these proteins is a beta-barrel, composed of antiparallel beta-strands. After synthesis in the cytoplasm, beta-barrel proteins reach the periplasm in an unfolded state, and are then folded and inserted into the outer membrane by the beta-barrel assembly machinery (BAM, fig. 1; Knowles et al. 2009; Hagan et al. 2011; Selkrig et al. 2013). The BAM complex in *Escherichia coli* consists of five subunits, BamA–E (fig. 1). BamA is essential (Genevrois et al. 2003; Voulhoux et al. 2003) and is found in all Gram-negative bacteria (Heinz and Lithgow 2014). As a member of the Omp85 protein family, BamA is itself a membrane-embedded beta-barrel protein (Noinaj et al. 2013) with several periplasmic polypeptide transport-associated (POTRA) domains (fig. 1). BamD, the other component essential in *Proteobacteria*, has also been identified in other groups of bacteria (Anwari et al. 2012; Webb et al. 2012), whereas other components of the BAM complex in *E*. *coli*, BamB, BamC, and BamE are outer membrane lipoproteins found only in subsets of the *Proteobacteria* (Anwari et al. 2012; Webb et al. 2012).

The sequenced genomes of *E. coli* and closely related bacteria such as *Citrobacter rodentium* encode a second member of the Omp85 protein family, TamA, which is part of a newly identified protein complex referred to as the translocation and assembly module (TAM, fig. 1; Selkrig et al. 2012). The second component of the TAM is TamB, a large enigmatic protein of unknown structure, which is encoded in an operon alongside the gene for TamA (fig. 1). The TAM has been demonstrated as necessary for the efficient assembly of several autotransporter proteins (Selkrig et al. 2012; Shen et al. 2014); it is not essential in the organisms studied so far, but its deletion eliminated the virulence or colonization potential in *C. rodentium, Salmonella enterica* (Selkrig et al. 2012), *Klebsiella pneumoniae*, *Proteus mirabilis* and *Vibrio fischeri* (Struve et al. 2003; Burall et al. 2004; Brooks et al. 2014), highlighting it as an attractive target for antimicrobial compounds.

We recently demonstrated that the TamA protein is present in most *Proteobacteria* but is not universal among Gram-negative bacteria, with additional subfamilies of Omp85 proteins encoded as well as, or instead of, TamA in some bacterial genomes (Heinz and Lithgow 2014). The distribution of the TamB protein has not been analyzed, but initial observations noted the occurrence of TamB in *Borrelia burgdorferi*, a member of Class *Spirochaetes* that lack TamA (Selkrig et al. 2012). Given that the nonessential partner subunits of the BAM complex can vary between different bacterial lineages and even between Classes as observed within the *Proteobacteria*, we examined the co-occurrence of TamB and TamA across diverse bacterial lineages. In so doing, a similarity between the TamB protein family and a family of proteins that included AsmA was noted based on sequence similarities as well as common structural traits. Like TamB, AsmA plays a role in outer membrane assembly (Misra and Miao 1995) and is implicated in virulence in *S*. *enterica* (Prieto et al. 2009). The canonical TamB from *E. coli* has been shown to carry a signal-anchor domain; that is, a hydrophobic stretch at the N-terminus which serves as a helical transmembrane segment integrated in the bacterial inner membrane (Selkrig et al. 2012). The rest of the protein extends into the periplasm with an extensive beta-helix fold that contributes toward an elongated shape (Josts et al. 2014; Shen et al. 2014). Due to the importance of the outer membrane protein assembly machineries for our basic understanding of how outer membrane biogenesis takes place, as well as the interest of TAM as a therapeutic target specifically targeting the virulence potential of Gram-negative cells, we performed a comprehensive analysis on the diversity and evolution of the TAM complex with a focus on TamB.

## Materials and Methods

### Databases and Software Packages

All searches were performed against, and sequences and taxonomic information were retrieved from, the UniProt database (Magrane and Consortium 2011; release 06032013) unless stated otherwise. Protein domains were retrieved from the Interpro database (Hunter et al. 2012; version 41.0). MCL was performed using the mclblastline suite (mcl version 12-135; Enright et al. 2002), with several different inflation parameters, where the optimal settings were chosen after manual inspections; all-against-all BLAST values for mclblastline clustering were obtained by using the BLASTall -p BLASTp command (BLASTall 2.2.24) with the -m8 output option and an *e* value cutoff of 1E-1, all other settings as default. For network representations in cytoscape (version 3.1.1; Shannon et al. 2003), protein diversity was first reduced by clustering all sequences with the usearch program (Edgar 2010; search performed using the -cluster_fast algorithm with a cutoff of -id 0.XX as given in the respective figure legends; the –centroid command was used to obtain the sequences). The resulting sequences were used as input for an all-against-all BLASTp run (version 2.2.26+; cutoff as indicated in the respective figure legends) and self-loops were removed before network analyses, the respective algorithms are given in the figure legends. Secondary structure predictions were performed using Phyre2 (Kelley and Sternberg 2009), BetaWrapPro (McDonnell et al. 2006) and psipred (McGuffin et al. 2000) or, if the maximal sequence length was exceeded, DomPred (Bryson et al. 2007). Plots were generated with the R software package (R Development Core Team 2011; http://www.r-project.org/), and data parsing was performed using in-house R and Python scripts.

### Signal-Anchor Identification

Signal sequences were recovered using SignalP version 4.1 (Petersen et al. 2011) using the default settings and version 3.0 using the hmm method (Nielsen and Krogh 1998; Bendtsen et al. 2004). To test the validity of this combined approach, proteins annotated to contain a signal-anchor sequence were retrieved from SwissProt reduced to an identity of 0.9, using the following search term at the UniProt web interface (uniprot:(keyword: “Signal-anchor [KW-0735]” taxonomy: “Bacteria [2]” reviewed:yes) AND identity:0.9); retrieved on February 17, 2015. These sequences were subjected to SignalP 3.0 and SignalP 4.1 as described in the Results section, and revealed a prediction of 274 of 307 input sequences under the described criteria (supplementary table S3, Supplementary Material online; lower part - UniRef90 accession numbers) and were subsequently used for TamB signal-anchor predictions (supplementary table S3, Supplementary Material online; upper part).

### Data Set Generation

The initial data set was generated based on a JackHMMER (v 3.0; Eddy 2011) run using the *E. coli* TamB sequence (UniProt: P39321) as input with the –max option and all other settings as default, running for five iterations. The protein domains of the respective hits were obtained from the InterPro matching table provided by the UniProt release (protein2ipr.dat), and domains of interest were plotted using the R software; all following analyses are based on the resulting hits from the fifth jackHMMER iteration. MCL was performed using inflation parameters 1.0, 1.1, 1.2, 1.5, 1.8, and 2.0. Following manual assessment, the clusters generated with inflation 1.2 (–blast-ecut = 1e-2 –mcl-scheme = 7 –mcl-I = 1.2) were used for further analyses. The clusters were manually annotated, and eukaryotic sequences as well as sequences less than 200 amino acids were removed from further analyses. To identify the domains associated with TamB-like proteins, and investigate whether the DUF490 domain was consistently located at the C-terminus, the locations of the annotated Pfam domain(s) for each protein in the clusters of interest were retrieved from the protein2ipr.dat table. All protein lengths were set to 100%, and the relative location of the respective domains for all proteins in the respective clusters are represented as cumulative histograms for each cluster (supplementary fig. S4, Supplementary Material online). For the heatmap demonstrating the presence or absence of BamA, TamA, TamL, and TamB, the UniProt hits were restricted to entries labeled “complete proteome,” and the number of organisms per Phyla with complete proteomes retrieved from the UniProt release; the data for BamA, TamA, and TamL were retrieved from Heinz and Lithgow (2014). For the prediction of the signal peptides, identical sequences were removed by using the uclust cutoff of 1.0.

### POTRA and Barrel Selection

A subset (supplementary table S6, Supplementary Material online) of sequences was used for the in-depth analysis of the POTRA sequences and phylogenetic tree inference; the barrel was defined as the sequence starting after the last POTRA until the C-terminus of the respective proteins. The POTRA domains for conserved BamA proteins with five POTRA domains were predicted based on their alignment to the sequence A1KRL4_NEIMF, which was aligned with, and the POTRA domains defined based on, the BamA *Neisseria gonorrhoeae* structure (Noinaj et al. 2013); the alignment with structural information was obtained through phyre2. The POTRA domains for the TamA and TamL data set were obtained based on the previous study on the Omp85/TpsB superfamily (Heinz and Lithgow 2014). Proteins with additional or reduced numbers of POTRA domains to their canonical homolog (e.g., BamA sequences with more or less than five POTRA domains) were annotated manually, based on results from the online predictors psipred and phyre2. If the secondary structure motif did not match perfectly, but the sequence was either embedded between clearly identifiable POTRA domains, or sequence without secondary structure prediction was present in the region where the missing secondary structure element would be expected, the sequences were also considered putative divergent POTRAs. Equally, POTRA candidate sequences were allowed if a short insertion in a loop between the POTRA structural elements could be observed.

### Phylogenetic Tree Inference

Alignments were generated with mafft (version 7.164b; Katoh and Standley 2013) using the -linsi option or muscle (Edgar 2004) as implemented in Seaview (version 4; Gouy et al. 2010), and sites for tree inference were chosen using trimal under the “-automated1” setting (Capella-Gutierrez et al. 2009). Trees were calculated using MrBayes with a mixed amino acid model (version 3.2.1; Ronquist et al. 2012) and RaXML (version 7.2.8; Stamatakis 2006) with the rapid bootstrap analysis, 100 bootstrap iterations and the “PROTGAMMALGF” model. MrBayes trees were calculated for 5 or 10 million generations as indicated in the figure legends under the mixed amino acid model reaching a final AvgStdDev of 0.012 or smaller, burn-in was set for 25%. Best model fit was investigated prior to running RaxML using ProtTest (version 3.1; Darriba et al. 2011) with the -all-matrices -all-distributions options, and resulted with the LG matrix (Le and Gascuel 2008) as best matrix for all data sets. Tree representation was performed using the itol tool (Letunic and Bork 2011). For the tree inference of TamB, sequences under 1,000 amino acids were removed to improve alignment quality. The sequence alignments and tree files have been deposited on FigShare. (http://figshare.com/articles/Evolution_of_the_Translocation_and_Assembly_ Module_TAM_/1424665, last accessed June 3, 2015.)

### Motif Analyses

To retrieve the C-terminal 15-amino acid TamB sequences, the full-length sequences were clustered using the uclust program with a clustering setting of id -0.90; the resulting centroids were then used to retrieve the most C-terminal 15-amino acid sequences from each protein, and were used as input for logo formation. The logo representation was performed using the online server seq2logo (version 2.0; Thomsen and Nielsen 2012) with the settings at P-Weighted Kullback–Leibler and Clustering (Hobohm1) and Weblogo default colors. For the C-terminal analysis of outer membrane proteins, the proteomes of the organisms as given in supplementary table S7, Supplementary Material online, were retrieved from UniProt. Beta-barrel prediction was performed using mcmbb (Bagos et al. 2005) and the cellular location was predicted using psortb (Bagos et al. 2005; Yu et al. 2010) with the settings for Gram-negative bacteria. The data set displays a positive value from mcmbb as a cutoff for integral outer membrane barrels as suggested by the authors, and a psortB prediction of “Outer Membrane.”

## Results

### TamB-Like Proteins Are Widespread

**Fig. 2.—:**
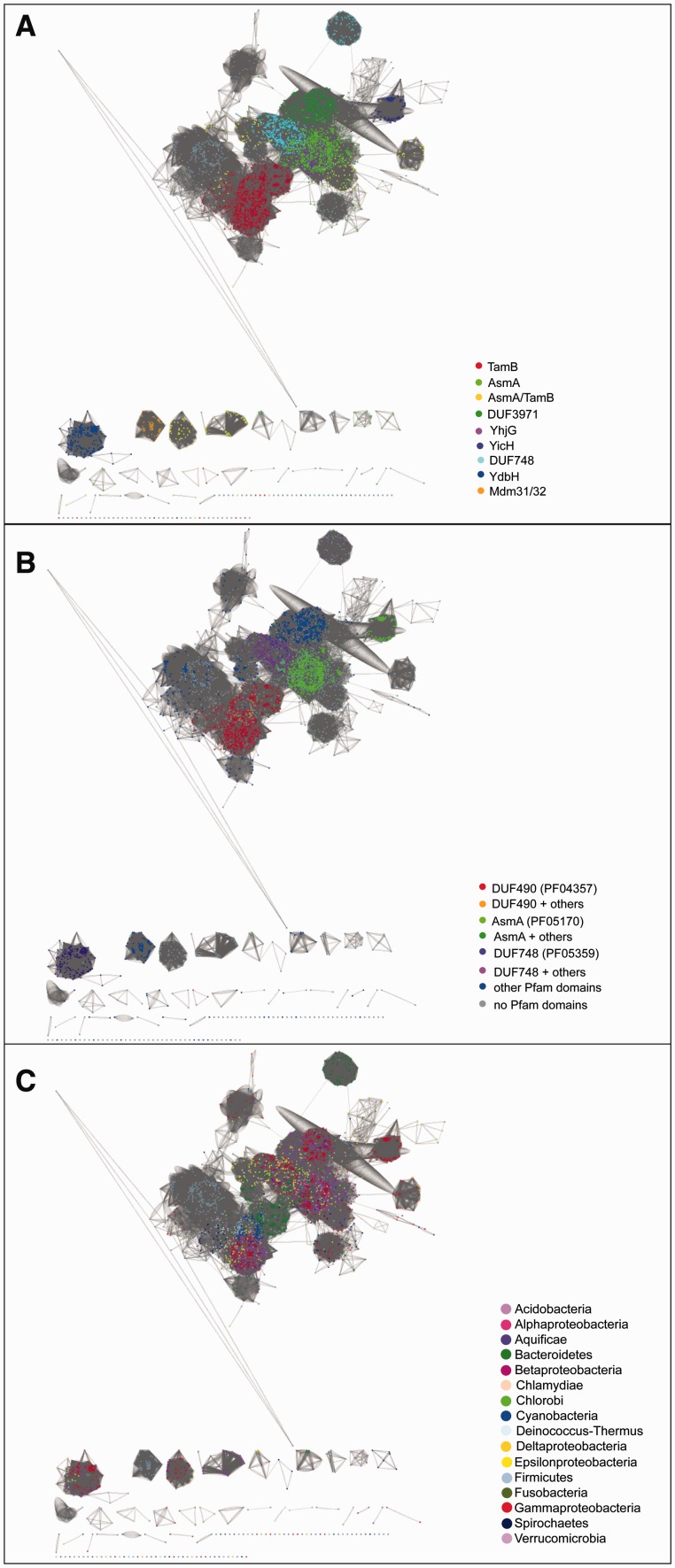
Relationships of all sequences after the last JackHMMER iteration. Clustering analysis of TamB and similar proteins shows its similarity to AsmA, as well as several other characterized and uncharacterized proteins. The displayed sequences were reduced to identity 0.9, the edges represent all-against-all BLAST *e* values with a cutoff of 1e-5, and the network visualization shows a force-directed network weighted on the edges. The colors represent the clusters as manually assigned in (*A*), in (*B*) the Pfam domains annotated for the respective sequences, and in (*C*) the taxonomic group of the sequences as indicated in the respective legends.

Initial hidden Markov model (HMM) searches (HMMER) of TamB highlighted a similarity to AsmA. Given the low overall similarity of TamB sequences we observed, and the unclear assignment of several sequences as either TamB or AsmA, we chose a very lenient cutoff to include as many divergent TamB-like sequences as possible for in-depth analysis. Domain analysis of the sequences identified in five iterative HMM searches (jackHMMER) using the *E. coli* TamB protein (UniProt: P39321) as the initial search input, showed that the number of proteins containing the DUF490 domain characteristic for TamB (Selkrig et al. 2012), as well as the AsmA domain, each plateaued at the fifth iteration (supplementary fig. S1, Supplementary Material online). Markov clustering (MCL) was then applied to the full set of 16,908 sequences after the fifth iteration (fig. 2). We note here a similarity between members of the TamB and AsmA protein families and the mitochondrial inner membrane proteins Mdm31 and Mdm32 (Dimmer et al. 2005), whether this is of functional significance awaits further investigation. The resulting subclusters were grouped following manual annotation, as well as removing fragments (<200 amino acids) and eukaryotic proteins resulting in 16,107 sequences (supplementary table S1, Supplementary Material online). Clustering and manual analyses also revealed the similarity of TamB not only to AsmA (including the two *E. coli* AsmA paralogs, YicH and YhjG) but also to a group of protein sequences with DUF748 and DUF3971 domains of unknown function (fig. 2 and supplementary fig. S2, Supplementary Material online). This relationship was confirmed in a comparison of the respective Pfam motifs using a pairwise comparison of profile HMMs (HHPred; http://toolkit.tuebingen.mpg.de/hhpred (last accessed October 11, 2014); Soding et al. 2005; supplementary table S2, Supplementary Material online): There is an underlying similarity of the Pfam domains between DUF490 (found in TamB) and AsmA, Mdm31/32, DUF3971, and DUF748. The protein clusters were manually joined into groups; despite several attempts at clustering and phylogenetic analyses, some sequence groups could not unambiguously be grouped with TamB or AsmA, and are therefore labeled as AsmA-TamB hereafter. The proteins grouped as TamB predominantly showed distinct sequence relationships according to their taxonomic species of origin (fig. 2 and supplementary fig. S2, Supplementary Material online).

**Fig. 3.—:**
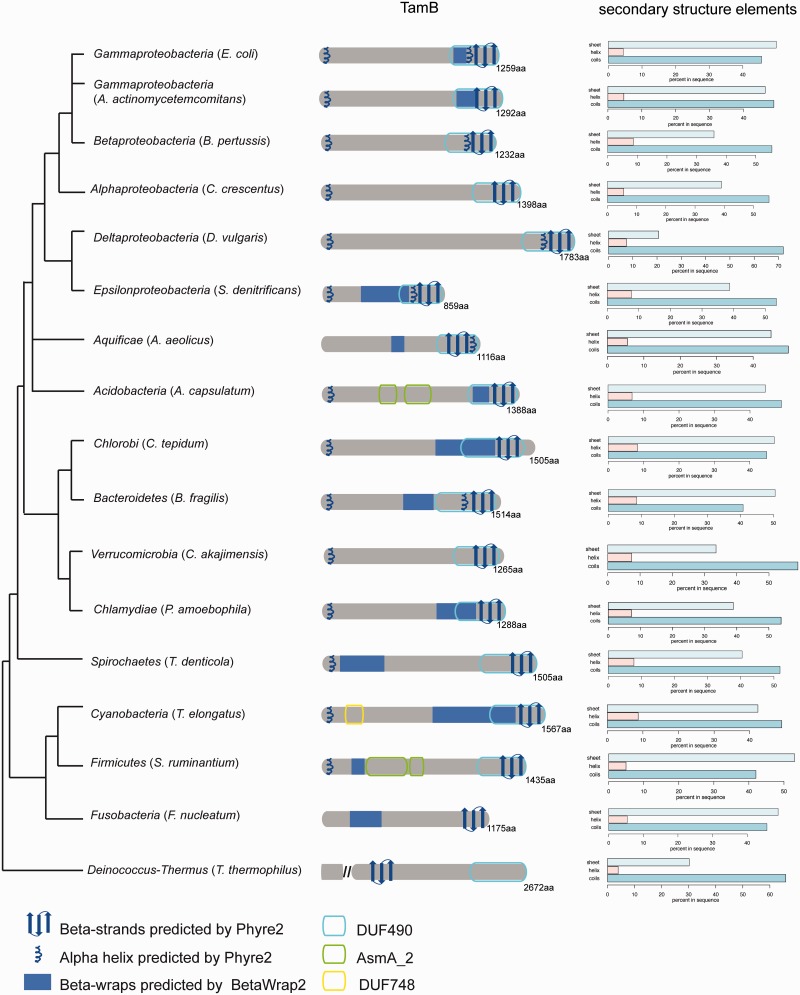
Conserved secondary structure features of TamB. Selected TamB sequences from the organisms indicated are shown as diagrams, and secondary structure prediction results are indicated, the right panel shows the percentages of helices, sheets or coils as predicted by psipred for the respective TamB sequence. The taxonomic guidance tree is adapted from (Errington 2013). The location of the *Acidobacteria* was modified to more closely resemble the phylogenetic pattern observed in the canonical BamA sequences; the *Aquificae* were placed at the root of the *Proteobacteria* although their true position is uncertain due to extensive HGT in this group of organisms (Eveleigh et al. 2013).

The protein sequences defined as TamB show a relatively consistent length profile, with only few exceptions of very long length (supplementary fig. S3, Supplementary Material online). A characteristic feature of these sequences is a C-terminally located DUF490 domain (fig. 3 and supplementary fig. S4, Supplementary Material online). Those sequences classified as TamB, but with no Pfam-defined domains recognized with the given cutoff (fig. 3), have predicted secondary structure features that suggest a conserved structure (fig. 3). In *E. coli*, the DUF490 domain corresponds to a 37-kDa region within TamB (residues 914–1,259), and the number of residues in the DUF490 domains in other TamB sequences is relatively conserved. In contrast, AsmA proteins show more diversity regarding sequence length (supplementary fig. S3, Supplementary Material online), and the AsmA domain covers either the entire protein or only the N-terminal region (supplementary fig. S4, Supplementary Material online).

In summary, a group of TamB protein sequences can be defined with rigorous criteria, and these proteins were subjected to further sequence scrutiny.

### Inferred Structural Features of TamB

With this extensive data set of TamB sequences, we sought to investigate their conserved characteristic features. The canonical TamB from *E. coli* has been shown to carry a signal-anchor domain (Selkrig et al. 2012), anchoring it in the inner membrane. Prediction of signal-anchor domains is not straightforward as they are often misinterpreted as cleaved signal peptides (which would indicate an outer membrane or periplasmic location; Nielsen et al. 1999). An HMM designed to distinguish these from cleaved signal peptides was implemented in the eukaryotic version of SignalP3.0 (Nielsen and Krogh 1998; Bendtsen et al. 2004), and the latest version of SignalP 4.1 (Petersen et al. 2011) was significantly improved to distinguish transmembrane regions (and therefore signal-anchor domains) from cleaved signal peptides; the prediction of the latter is the goal of SignalP. We therefore combined SignalP 3.0 and SignalP 4.1 predictions, using results positive in SignalP 3.0 under the HMM (either predicted as secreted for bacteria or membrane-anchored for eukaryotes) and negative in SignalP 4.1 as a positive signal for a signal-anchor domain. A test of proteins annotated as signal-anchored in UniProt confirmed this strategy (for more details, see the Materials and Methods section). We can conclude that this seems to be a conserved feature of TamB: A majority of sequences (1,110 of 1,367) yielded prediction results as described above (supplementary table S3, Supplementary Material online), and an N-terminal helix is predicted for several sequences based on structural similarity (fig. 3).

The first reports on TamB assumed it to be a putative substrate of a two-partner secretion system, as secondary structure analyses clearly highlight an extensive region of the protein to encode beta-strands which are typical structural hallmarks of two-partner secretion system substrates (Stegmeier et al. 2007; Chaudhuri et al. 2010). In other studies, TamB was classified as an outer membrane protein, again because of beta-strand predictions (Diaz-Mejia et al. 2009; Babu et al. 2011). To better understand the secondary structure of TamB and its conservation through distant bacterial Phyla, we performed secondary and tertiary structure predictions using psipred, betawrap and phyre2 on sequences from a broad taxonomic distribution (supplementary tables S4 and S5, Supplementary Material online), including a protein called MorC in *Aggregatibacter actinomycetemcomitans* (UniProt: Q4JI69) which grouped with the largest cluster of TamB homologs (supplementary table S1, Supplementary Material online), and has structural characteristics of a TamB family member (fig. 3). MorC was identified in a screen for factors effecting membrane morphology (Gallant et al. 2008).

Betawrap is a tool that scores the match of a sequence to a right-handed beta-helix fold. Predictions of a beta-helical structure yield similar values to structurally defined autotransporters with large extended beta sheets (Drobnak et al. 2015; supplementary table S5, Supplementary Material online) in most sequences (fig. 3). The distribution of secondary structure elements according to psipred indicated predominantly beta-sheets and coils as summarized in figure 3 (right panel). The structural prediction server Phyre2 compares submitted sequences with known structures in the Protein Databank (Berman et al. 2000; Kelley and Sternberg 2009). Although the majority of the TamB protein shares no significant similarity to published structures at this level, the very C-terminal part consistently yielded hits with beta-barrel protein structures. This in turn suggests that the very C-terminal portion of TamB could form several amphipathic beta strands (fig. 3), akin to the beta-strands of outer membrane proteins. This observation on the TamB C-terminus was followed up by more detailed investigations of its sequence.

### Characteristic Motifs

Sequence logos were constructed for the C-terminal 15 amino acids of the TamB proteins for each bacterial Phylum (splitting the *Proteobacteria* into the five main classes). In most cases, a conserved phenylalanine (F) or tryptophan (Y) residue was apparent at the very last position or in very close proximity (supplementary figs. S5 and S7, Supplementary Material online). This was of interest given that the same residues have been proposed as essential for targeting beta-barrel proteins to the outer membrane (Struyve et al. 1991; Robert et al. 2006). The most prominent exception was seen in the *Bacteroidetes* and *Chlorobi*, where a very strong tendency for charged residues throughout the C-terminal amino acids was observed (supplementary fig. S5, Supplementary Material online).

Other groups, including the *Gammaproteobacteria*, gave no clear motif. As this could be a reflection of the high coverage of sequences available for members of the *Gammaproteobacteria,* we split the Class further into the respective Orders (supplementary fig. S6, Supplementary Material online). Although several Orders, for example, *Legionellaceae*, display a tendency for charged residues other groups, such as the *Enterobacteriaceae*, display a similarity in the logos for TamB sequences and outer membrane protein sequences. In particular, an enrichment of phenylalanine (F) at the last residue was observed (supplementary fig. S7, Supplementary Material online). To bring these observations of strong variations in the TamB C-terminus into context, we assessed the extent to which the C-terminal residue in outer membrane proteins is conserved in different organisms, which has predominantly been studied for *E. coli* and closely related species.

Beta-barrel outer membrane proteins for members of several distantly related Phyla, to cover a wider range of the bacterial diversity, were predicted starting from all annotated open region frames for the respective organisms (supplementary fig. S7, Supplementary Material online), and logos were constructed for the C-terminal 15 amino acids from these protein sets. This indicates that the C-terminal F-enrichment of outer membrane beta-barrel proteins has not undergone taxonomic adaptations: The only organisms with a lower level of conservation are members of the *Spirochaetes*, where 1) the TamB C-terminus shows an F/Y enrichment; and 2) even though less pronounced, F/Y are still enriched at the very C-terminus of outer membrane proteins from these organisms (supplementary fig. S7, Supplementary Material online).

### TamB Distribution, and Codistribution with Omp85 Family Members

**Fig. 4.—:**
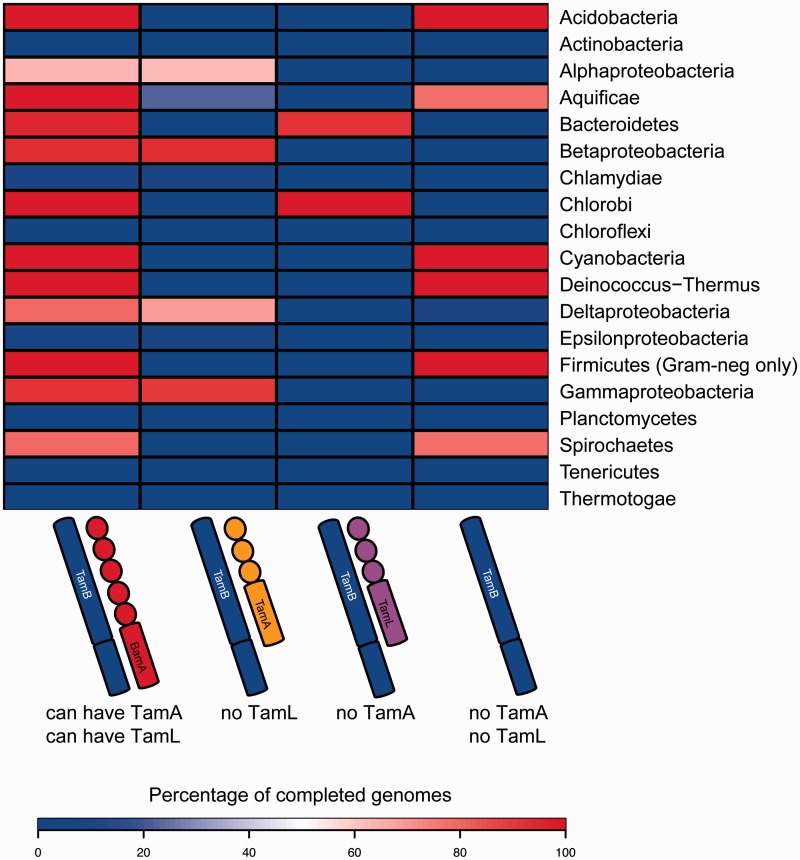
The distribution of TamA, TamB, TamL, and BamA. Percentages of all completed genomes of the respective Phyla, which encode for the indicated proteins, are shown by color shading: Blue indicates 0%, white indicates 50%, and red indicates 100% as indicated in the legend. The completed genomes of three organisms with outer membranes were found to lack BamA (Heinz and Lithgow 2014): *Coxiella burnetii* (strain CbuG_Q212) lacks BamA but encodes a TamA, and *Helicobacter pylori* (strain SouthAfrica7) and *Helicobacter pylori* (strain Gambia94/24) both lack any Omp85 proteins based on their genome data. The *Coxiella* strain encodes a TamB, whereas both *Helicobacter* strains encode an AsmA_TamB protein. Only Phyla (Classes for the *Proteobacteria*) with more than five taxa with completed proteomes according to UniProt are shown. For the *Firmicutes*, only the organisms with a Gram-negative like cell envelope are shown.

**Fig. 5.—:**
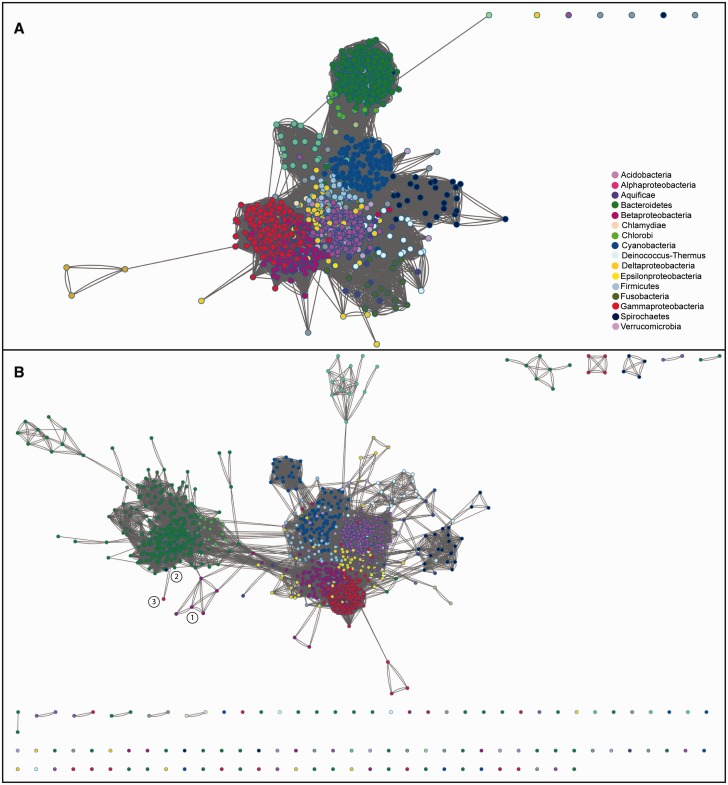
The sequence diversity of TamB. TamB sequences mostly cluster according to their taxonomic group as indicated by colors; (*A*) represents full-length TamB sequences, the displayed sequences of TamB and related proteins were reduced to identity 0.9, the edges represent all-against-all BLAST *e* values with a cutoff of 1e-5, and the network visualization shows a force-directed network without weight. (*B*) represents only the 50 most amino-terminal sequences of the same sequences as in (*A*); the edges represent an *e* value cutoff of 1e-1 and the visualization is force-directed without weight. Several exceptions indicated in (*B*) are discussed in the text.

A surprising result was the finding of such a broad distribution of TamB sequences, given the restriction of TamA proteins predominantly to the *Proteobacteria* (Heinz and Lithgow 2014). To interrogate the extent of codistribution of TamB and TamA, we generated a heatmap of all complete proteomes with a color gradient indicating the percentage of encoded respective combinations of proteins, irrespective of whether they are coencoded in an operon (fig. 4). This shows that TamB can be found in all bacterial Phyla with an outer membrane, with a few exceptions like the early-branching Phyla *Thermotogae* and *Thermodesulfobacteria*. It should be noted that the representation of TamB within any given Phylum is not complete, emphasizing that TamB does not have an essential function (fig. 4 and 5*A*).

TamB often co-occurs with TamA or the TamA-like Omp85 lipoprotein (Heinz and Lithgow 2014) found in the *Bacteroidetes* and *Chlorobi*. We designate this Omp85 lipoprotein now as TamL: As evidenced below, it occurs in operons with TamB, has high sequence similarity to, and likely shared common ancestry with, TamA, and occurs only in species that have no other copy of TamA encoded in their genomes. However, TamB also occurs in a noticeable group of bacteria without either TamA or TamL. To further investigate operon structures and secondary structure details of TamB, as well as the evolutionary relationships between the TamA, BamA and TamL proteins, we focused our analyses on a small data set of representative, taxonomically diverse species (supplementary table S6, Supplementary Material online).

**Fig. 6.—:**
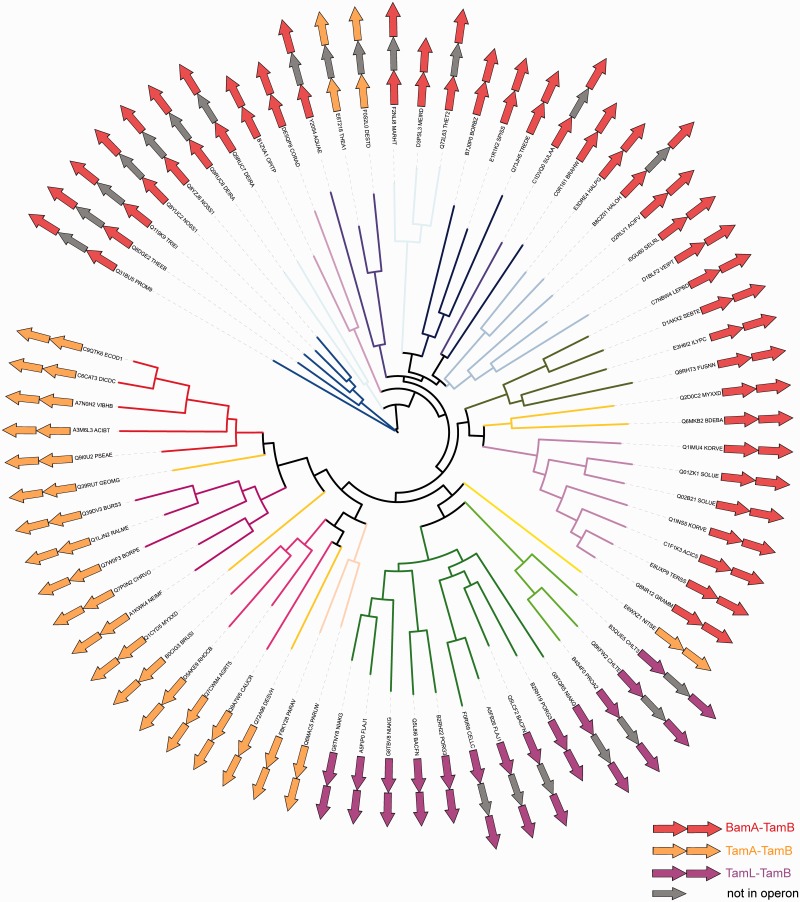
Phylogenetic analysis of TamB. The branches indicate the taxonomic lineage with the same color scheme as shown in the legend for figure 2*C*, potential operons are indicated in the pictograms. Red, BamA operon; orange, TamA operon; pink, TamL operon. Operons intercepted by a gray icon indicate the presence of an additional Omp85 on the genome, but not in an operon with TamB. The tree was calculated using RAxML as described in the Materials and Methods. An unrooted display of this tree including bootstrap support values is given in supplementary figure S8*A*, Supplementary Material online.

**Fig. 7.—:**
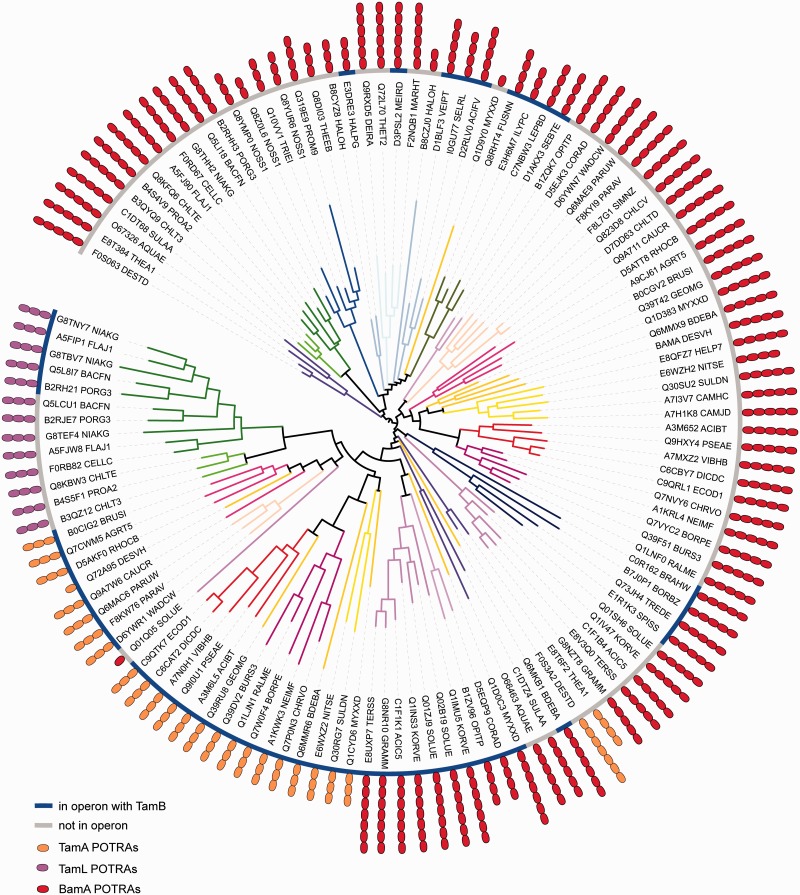
Phylogenetic analysis of Omp85 proteins. The BamA, TamA, and TamL sequences of organisms as given in supplementary table S6, Supplementary Material online, were used in a phylogenetic tree calculation. The branches indicate taxonomic lineage with the same color scheme as shown in the legend for figure 2*C*, the icons represent the number of POTRA domains and the nature of the protein (red, BamA; orange, TamA; pink, TamL). The ring indicates if the proteins are encoded in an operon with TamB; where gray indicates no operon, and blue indicates an operon with TamB. The tree was calculated using RAxML as described in the Materials and Methods. An unrooted display of this tree including bootstrap support values is given in supplementary figure S9*A*, Supplementary Material online.

**Fig. 8.—:**
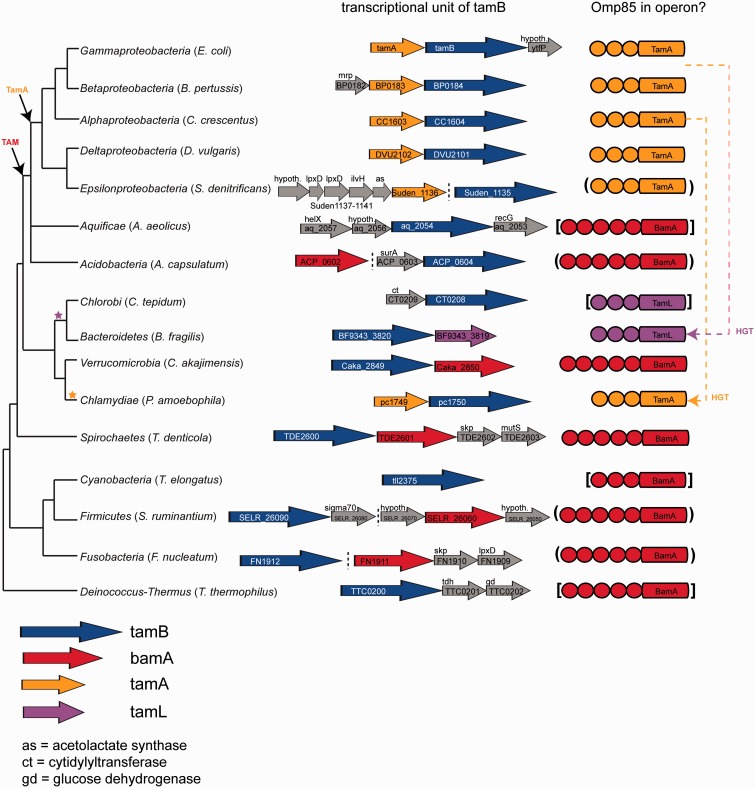
The evolution of the TAM operon. The left panel shows transcriptional units of *tamB* homologs as given in the BioCyc database collection (Caspi et al. 2014), the right panel highlights the presence or absence of an Omp85 protein in a putative operon with the respective TamB sequence. The orange dashed arrow indicates the likely HGT from the *Alphaproteobacteria* to a subgroup of *Chlamydiae*, as also seen in figure 6 and supplementary figure S9, Supplementary Material online; the orange/magenta dashed arrow indicates an HGT event of TamA from the ancestor of the *Beta*- and *Gammaproteobacteria* to the ancestor of the *Bacteroidetes*/*Chlorobi*, which acquired the lipid anchor and became TamL. Stars at the guidance tree reflect these two HGT events. For several genes, a gene encoding an Omp85 protein is present in the vicinity, but not in the same transcriptional unit. In the absence of experimental verification, these were interpreted as putative operons and are indicated by a dashed line separating the operons (left panel) and by round brackets surrounding the Omp85 protein icon (right panel). Protein icons enclosed by square brackets indicate the Omp85 protein most likely to interact with the respective TamB, but encoded elsewhere on the genome. The taxonomic guidance tree was derived as described for figure 3.

Phylogenetic analysis of TamB revealed no clear correlation of the TamB proteins with respect to their presence in an operon with an Omp85 protein besides the monophyletic origin of the sequences associated with TamA or TamL (fig. 6 and supplementary fig. S8, Supplementary Material online). The chlamydial TamA–TamB operon seems to be derived through horizontal gene transfer (HGT) from the *Alphaproteobacteria*; this is also supported in the analysis of the Omp85 proteins (fig. 7 and supplementary fig. S9, Supplementary Material online). Although the *Proteobacteria* TamB forms a large monophyletic cluster (with the *Chlamydiae*; fig. 6 and supplementary fig. S8, Supplementary Material online), several *Deltaproteobacteria* and *Epsilonproteobacteria* cluster with the early-branching Phyla (such as *Fusobacteria* and *Bacteroidetes*); this likely HGT event is also reflected in the Omp85 sequences (Heinz and Lithgow 2014; fig. 7 and supplementary fig. S9, Supplementary Material online). The *Acidobacteria* and *Aquificae* both encode a second copy of BamA, which branches off monophyletic with TamA, indicating this to be the putative origin of the TAM (figs. 7 and 8 and supplementary fig. S9, Supplementary Material online). A consistent clustering of the early-branching Phyla including *Spirochaetes*, *Deinococcus-Thermus*, *Firmicutes,* and *Cyanobacteria* was also observed for TamB.

TamB sequences show no indications for an overall sequence adaptation to its interaction partners: The sequences cluster together based on their taxonomic origins and not based on their occurrence in an operon with either BamA or TamA (fig. 5*A*). This is further emphasized when analyzing only the 50 most C-terminal amino acids of the TamB sequences (fig. 5*B*), where we note several interesting groupings: A set of *Betaproteobacteria*, the species *Cupriavidus* sp., clusters strongly with the *Bacteroidetes* instead of the other *Betaproteobacteria* sequences (fig. 5*B*, group 1), as do *Spirochaeta africana* and a *Pseudomonas* sp. sequence cluster with the *Bacteroidetes* (figs. 2, 3, and 5*B*); and several groups of *Deltaproteobacteria* can be observed (fig. 5). These examples suggest that HGT of TamB, although rarely, can be observed.

### Omp85 Protein Sequence Diversity in the Light of Potential TamB Interactions

Sequence similarities between Omp85 proteins likely interacting with TamB (e.g., BamA encoded in an operon with TamB, TamA, or TamL) were compared with those unlikely to interact with TamB (e.g., BamA in organisms with a TamA–TamB operon). Phylogenetic tree inference of the barrel domain (fig. 7 and supplementary fig. S8, Supplementary Material online) indicates that there are two major groups including putative TamB-interacting proteins in the organisms investigated. One group is comprised TamA (*Proteobacteria*), TamL (*Bacteroidetes* and *Chlorobi*), and BamA sequences closely related to the *Proteobacteria*: Members of the *Aquificae* and *Acidobacteria*. The other major group comprises the early-branching Phyla (*Cyanobacteria*, *Firmicutes*, *Deinococcus-Thermus*) and is likely the root of the BamA proteins; this is also reflected when considering a tree of only BamA sequences (supplementary fig. S10, Supplementary Material online). The third major group observed is not likely to interact with TamB, and consists of the *Proteobacteria*, *Chlamydia*, and *Acidobacteria* canonical BamA proteins (fig. 7 and supplementary fig. S8, Supplementary Material online). Several of the proteins potentially interacting with TamB encode for more POTRA domains than the canonical BamA (fig. 7), and some of these POTRAs show clear divergence from conserved BamA POTRAs as discussed in the next paragraph. Taking together the information from the phylogenetic analysis of TamB, Omp85 proteins, and the current understanding of the evolution of bacterial Phyla, the TAM likely occurred for the first time in the shared common ancestor of *Proteobacteria* and *Acidobacteria*/*Aquificae*. It should be noted that the placement of the *Aquificae* in the bacterial evolution is uncertain (Eveleigh et al. 2013); the presence of the TamA-like BamA in these organisms could also be the result from a HGT event from the *Acidobacteria*, should the true origin of the *Aquificae* lie closer to the root of the bacterial diversity. However, the *Aquificae* could also be a reflection of the transition from a duplicated BamA to TamA, where some of the duplicated BamA sequences show closer similarity to BamA, whereas others already cluster together with TamA (Heinz and Lithgow 2014; fig. 7 and supplementary fig. S9, Supplementary Material online). The origin of the *Bacteroidetes*/*Chlorobi* TAM operon comprised TamL–TamB is most likely the result of a HGT event from the common ancestor of the *Beta-* and *Gammaproteobacteria* (fig. 7 and supplementary fig. S9, Supplementary Material online); and a HGT from the *Alphaproteobacteria* is most likely the origin of the TAM operons in several chlamydial species (fig. 8).

POTRA sequences are very short, and we therefore used a clustering approach to assess whether distinct groups could be observed between POTRAs of proteins likely interacting with TamB compared with the ones that likely do not interact. The strong divergence of POTRAs 1 and 2 of TamA when compared with BamA POTRAs was recently reported (Heinz and Lithgow 2014), and we sought to expand this knowledge by including the BamA POTRA domains from our restricted sequence selection, taking into account whether or not these co-occurred with TamB. This was of particular interest given that several proteins had more than the classical five POTRA domains for BamA (fig. 7). The clustering showed that although POTRAs at the termini of these BamA proteins are relatively conserved, several sequences for putative internal POTRAs (POTRA 3 or 4 from proteins encoding seven or six POTRAs, respectively; supplementary fig. S11, Supplementary Material online) show a divergence comparable to POTRA 2 from TamA and POTRAs 1 and 2 of TamL. Although this might suggest a diversification of the POTRA domains under selective pressure from interaction with TamB, it needs to be considered that the secondary structure prediction for these POTRAs was not perfectly aligned with the usual β-α-α-β-β profile as outlined in the legend of supplementary figure S11, Supplementary Material online. It is therefore unclear whether these sequence segments are genuine POTRA domains, or might instead represent novel, long intervening regions rendering more flexibility to the respective POTRA arms. Interestingly, these POTRA domains are found on the *Acidobacteria* BamA likely representing the origin of the TAM, as well as on the BamA of the *Deinococcus-Thermus* group, which encode for a TamB and only one BamA (fig. 7).

## Discussion

The BAM and TAM machineries are key to outer membrane biogenesis. The BAM complex consists of one Omp85 protein, BamA, which interacts with its binding partners through its periplasmically located POTRA domains. These interaction partners however are not conserved and BAM subunits vary within and between different bacterial taxa (Webb et al. 2012). In light of the evolution of the TAM, TamA was known to be almost exclusively confined to the *Proteobacteria* (Heinz and Lithgow 2014). Our first goal was therefore to carefully define sequence criteria to identify members of the TamB family: This revealed a distribution across almost all bacterial Phyla with an outer membrane, clearly exceeding the distribution of TamA. In stark contrast, we could not observe any occurrence of TamA in species where no TamB was encoded on the genome.

As summarized in figure 8, our analyses revealed a significant part of the bacterial diversity encoding *tamB* and *bamA*—without *tamA* or *tamL*—and often together in an operon. This could indicate the formation of a BamA–TamB complex, or be a reflection of their functional relatedness, rendering them under the same transcriptional control but without further physical interactions: This is relevant given our observation of a potential relation between TamB and AsmA, given that AsmA is reported to play a role in outer membrane protein assembly with no evidence as yet that it operates together with an Omp85 partner (Misra and Miao 1995; Deng and Misra 1996; Xiong et al. 1996).

What of the alternative scenario, wherein TamB interacts with BamA in these organisms? There have been functional analyses of BamA from organisms with a TamB–BamA operon (Lenhart and Akins 2010; Lenhart et al. 2012), or a TamB encoded on the genome separately from BamA, but again without TamA in the genome (Nesper et al. 2008; Tripp et al. 2012; Estrada Mallarino et al. 2015). Although these studies have not reported BamA–TamB interactions, neither have they excluded the formation of a TamB–BamA complex or more transient interaction between the proteins. Future biochemical work will be required to directly address the hypothesis of a BamA–TamB interaction in these species of bacteria.

Secondary structure analyses across the TamB protein family revealed a high degree of conservation of the overall architecture: With an N-terminal signal anchor domain and a C-terminal DUF490 domain incorporating several beta-barrel-like strands at the very C-terminus. Relevant to its interaction with Omp85 partner proteins, the C-terminal amino acids mirror those of beta-barrel proteins with a high enrichment of F or Y at the last position, which is an essential feature for protein targeting to the outer membrane in *E. coli*. Although TamB is found in the inner membrane fraction of cells (Selkrig et al. 2012), the outer membrane beta-barrel protein characteristics of its C-terminus are striking. One possibility would be for TamB to mimic the substrates of TamA, and function as a pseudosubstrate bound to TamA until higher affinity substrates bind. In this speculative scenario, TamB would serve to regulate the activity of TamA. Indeed, a regulatory effect of TamB on TamA was observed in the biophysical behavior of the TAM interacting with a substrate (Shen et al. 2014).

Our phylogenetic analyses indicate that TamB was already present very early in bacterial evolution, and suggest that the first configuration of outer membrane assembly components was a combination of TamB and BamA, in organisms with only one canonical BamA in the genome (as seen today in the *Fusobacteria*, Gram-negative *Firmicutes*, and some members of *Deinococcus-Thermus*). A model for the evolution of the TAM postulates that duplicate copies of BamA, such as seen in the *Acidobacteria* and *Aquificae,* form the origin of TamA. Some of these duplicated BamA sequences encode seven POTRAs, where the third POTRA shows higher divergence from the conserved BamA POTRAs, indicated in supplementary figure S11, Supplementary Material online. These proteins subsequently acquired the characteristic POTRA domains of TamA and TamL as we see today (fig. 8). The position of the *Spirochaetes* in the phylogenetic analysis indicates at least two independent events for the formation of a BamA–TamB operon, which is also supported by their reversed order of TamB–BamA versus BamA–TamB found in the *Acidobacteria*. In addition, in the *Spirochaetes*, the protein in an operon with TamB is the canonical (and only) BamA with the characteristic five POTRA domains. This protein is branching off with either the canonical (supplementary fig. S9*A*, Supplementary Material online) or the duplicated (supplementary fig. S9*B*, Supplementary Material online) BamA from the *Acidobacteria* using either maximum likelihood or Bayesian tree inference, respectively; the grouping of the *Spirochaetes* BamA is therefore uncertain, but in contrast to the grouping of their TamB sequences, which cluster with the early-branching Phyla under both approaches (supplementary fig. S9*A* and *B*, Supplementary Material online).

The TAM was only identified very recently (Selkrig et al. 2012), and the known substrate spectrum is so far limited to several autotransporters, where the role of TAM in their assembly was demonstrated by biochemistry and biophysics (Selkrig et al. 2012; Shen et al. 2014). Autotransporters have a far from universal distribution in bacteria (Celik et al. 2012), so the finding that TamB has widespread occurrence also begs a question regarding the substrate spectrum of the TAM: There are no autotransporters in several of the Phyla encoding TamB (without TamA) or TamL and TamB, and even for the organisms encoding a TamA–TamB operon, it is unclear whether the encoded autotransporters are substrates of TAM (Leyton et al. 2012, 2014; Roman-Hernandez et al. 2014). What then is the basis for selection of the TAM across such a diversity of bacterial species? A recent study (Smith et al. 2015) suggests that TamB has a much broader set of substrates. The outer membrane proteome of a deletion mutant lacking TamB (MorC) was analyzed, revealing changes including several proteins involved in quality control systems, oxidative stress responses and toxin secretion (Smith et al. 2015), and the entire membrane morphology of the mutants was shown to be strikingly modified (Azari et al. 2013).

### Final Conclusions

An understanding of the TAM is just beginning, and our study contributes important information on its evolution, conserved structural features, and function in outer membrane biogenesis. We have shown that the distribution of TamA and TamB is the opposite of the distribution of the subunits of the BAM complex; with TamB being far more widely distributed than its Omp85 partner TamA. Like BamA, TamB was present very early in the evolution of bacteria, and its repeated occurrence in operons with Omp85 proteins indicates its tight functional link to outer membrane protein biogenesis across the bacterial diversity. Several secondary structure characteristics show a remarkable conservation and are characteristic for the TamB protein family. Detailed phylogenetic analysis allows formulation of a hypothesis for the evolution of the TAM: Although TamB was already present from the earliest-branching Phyla, TamA is the result of a gene duplication of BamA followed by drastic adaptation or partial sequence exchange of two POTRA domains, resulting in the TAM complex exemplified today in *E. coli*.

## Supplementary Material

Supplementary tables S1–S7 and figures S1–S11 are available at *Genome Biology and Evolution* online (http://www.gbe.oxfordjournals.org/).
